# The Medical Historical Cultural Foundations of Western Nasal Surgery from Ancient Greece to the Middle Ages

**DOI:** 10.1007/s00266-022-02989-2

**Published:** 2022-10-20

**Authors:** Silvia Marinozzi, Riccardo Carbonaro, Daniela Messineo, Edoardo Raposio, Luca Codolini, Giuseppe Sanese, Valerio Cervelli

**Affiliations:** 1 Department of Molecular Medicine, Unit of History of Medicine and Bioethics, Sapienza University of Rome, 34/a Viale dell’Università, 00161 Rome, Italy; 2 International Medical School Tor Vergata, University of Rome, Via Montpellier 1, 00133 Roma, Italy; 3 Department of Plastic and Reconstructive Surgery and School of Specialization on Plastic, Reconstructive and Aesthetic Surgery, School of Medicine and Surgery, Tor Vergata University of Rome, Via Montpellier 1, 00133 Rome, Italy; 4 Department of Radiological Sciences, Oncology and Anatomo-Pathological Science, Sapienza University of Rome, 31/33 Viale dell’Universitá, 00161 Rome, Italy; 5 Plastic Surgery Division, Department of Surgical Sciences and Integrated Diagnostics-DISC, University of Genoa, Largo R. Benzi 10, 16132 Genoa, Italy; 6 Unit of Plastic Surgery, Sapienza University of Rome, Viale del Policlinico 155, Rome, Italy; 7 PhD School on Medical-Surgical Applied Sciences-Plastic Regenerative research area, School of Medicine and Surgery, Tor Vergata University of Rome, Via Montpellier 1, 00133 Rome, Italy; 8 Private Practice, Rome, Italy; 9 Università degli Studi di Milano, Milano, Italy

**Keywords:** Rhinoplasty, History of medicine, History of surgery, History of rhinoplasty, Byzantine medicine, Hippocrates

## Abstract

**Abstract:**

The manuscript aims to clarify the origins of Western rhinosurgery through the ancient texts of the greatest physicians of the past, up to the Byzantine Era, focusing on the “exchange of knowledge” between peoples. This excursus is carried out by quoting the texts of the greatest doctors of the past, such as Hippocrates, Galen and Celsus and by analysing the works of Byzantine authors such as Oribasius, Aetius, Antillus, which, more than others, represent the moment of fusion and interpenetration of Ancient Medical knowledge, paving the way for the Medieval Scholae Medicae in the West. The aim, therefore, is to fill that sort of "great gap" (from the foundation of Constantinople in the 4th century AD to the early Arab culture in the 11th century AD) due to the fact that figures such as Branca, Vianeo and, finally, Tagliacozzi, are considered direct actors of a recovery of the “ancient knowledge” of classic authors. This literature tends to less evaluate, instead, that important and huge cultural exchange -literally osmotic- in medical and surgical knowledge between peoples and civilizations, that find a *trait d'union* in the application of medical knowledge and surgical practical techniques matured in the Byzantine, Arab and Early Medieval period. In final analysis, through the History of Rhinosurgery, this paper aims to highlight how Western medical knowledge is made up of the ensemble of cultures which are apparently distant and different from each other, which merge themselves in a truly universal and transcultural knowledge: the Medical knowledge.

**Level of Evidence V:**

This journal requires that authors assign a level of evidence to each article. For a full description of these Evidence-Based Medicine ratings, please refer to the Table of Contents or the online Instructions to Authors www.springer.com/00266.

## Introduction

Rhinoplasty embodies perfectly the dual nature, aesthetic and reconstructive, of Plastic surgery: in fact, the nose, being a central and distinctive anatomical structure of the face, can be considered at the same time an essential component of the facial eurythmics, while it also remains fundamental for respiration, sense of smell, and social interactions in general. Its central and prominent position on the face and its complex osteocartilaginous structure makes it vulnerable to traumas and damages since the early ages of civilization, such as in combat wounds, mutilations or in disfiguring pathologies.

The relative abundance of “surgical demand” concerning this organ, both for its functional and aesthetic role, has led Physicians to develop, since ancient times, reconstructive modalities capable to restore the “*status quo antea”* with increasingly refined procedures [1].

In this article we will proceed, assisted by ancient sources, to reconstruct the cultural foundations and the history of rhinoplasty techniques from antiquity to the Middle Ages. The aim of the authors is to write an historical-medical article describing the moment of fusion and interpenetration of Ancient Medical knowledge paving the way for the Medieval Scholae Medicae in the West and to clarify the origins of Western Rhinosurgery through the study of ancient texts and illustrations (albeit not coeval) of the greatest physicians of the past, from Roman authors up to the Byzantine Era, focusing on the less known authors and highlighting the “exchange of knowledge” between peoples: the paradigm at the base of modern scientific Knowledge.

## From India to Western Civilization: First Hints of Knowledge Exchange Between Peoples About Nasal Surgery

**Fig. 1:**
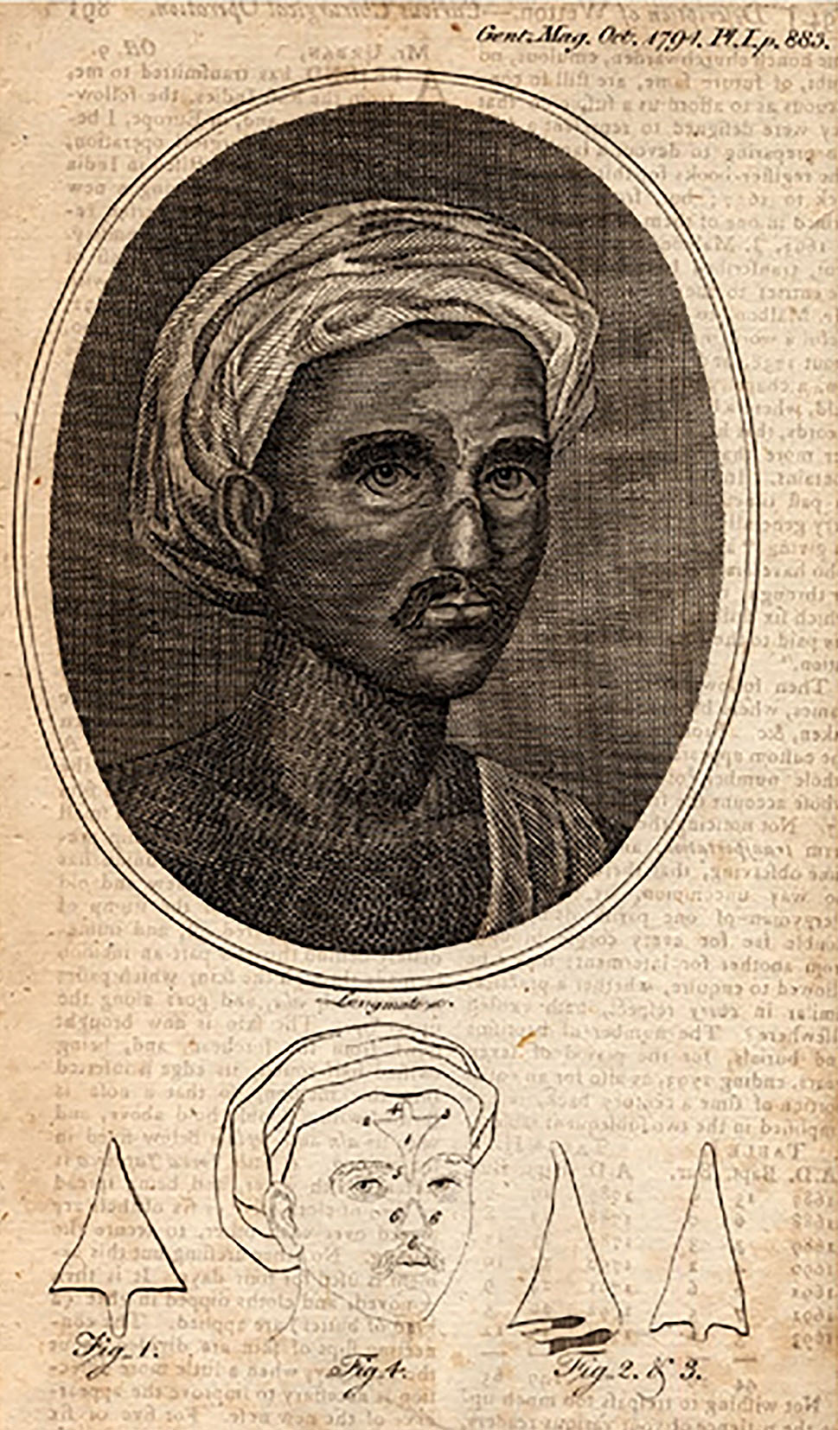
Indian technique for nose reconstruction and bandage: the Median Forehead Flap. (in The Gentleman’s Magazine and Historical Chronicle, Oct 1794, (Part the Second), 64, p. 883, Reference: Todd, W. B. “Bibliographical account of The Gentleman’s magazine, 1731–1754”, in: Studies in Bibliography. Vol. 18:81–109 (1965); DNB (1909 ed.), III:1248–1249; ESTC P1957; NCBEL, II:1295–1296; Times handlist, p. 40; Ward, W. S. Index of serials, p. 63. Wellcome Library-Wellcome journals. link: https://archive.org/details/s2492id1330028/page/n325/mode/2up. License: http://creativecommons.org/publicdomain/mark/1.0/)

It is common knowledge that Indian surgeons in the sixteenth century B.C. already practiced nasal reconstruction using systems based on flaps harvested from the forehead or cheeks to treat the results of the cruel punishments of rhinectomy inflicted to culprits of adultery [2–4] (Fig. 1)

In the West, we can find the first hints on Facial plastic surgery in the texts of Hippocrates (Greece, IV-V century B.C.), considered the founding father of Modern medicine since he was the first to separate the art of medicine from religion, moving toward an increasingly scientific approach based on a specific doctrinal body. He thus founded his humoral theory to explain the state of good health as an equilibrium between the elements of the body and, on the other hand, the disease as a state of imbalance of those elements.

Concerning the treatment of traumas and wounds, we can find many pathologic descriptions and treatment modalities in the Corpus Hippocraticum, which comprises sixty scripts, some of which written directly by Hippocrates and his students, other posthumous but still linked to his teachings [5]. Regarding the lesions of the nose and the therapeutic procedures for its reconstruction, the main source is indubitably his “*De articulationibus* and *De fracturi”*.

In the II century, Galen of Pergamon (II-III century A.D.), the main exponent of roman medicine, *summa* of ancient medicine, transcribed and commented the Hippocratic texts allowing to spread his techniques in the Roman Empire. Born in Pergamon, now in Turkey, Galen arrived in Rome as physician of the gladiators. His medical and philosophical knowledge and his exceptional skills brought him to become the Emperor’s physician for Marco Aurelio and his son Commodus.

His texts and his anatomic, physiologic and pathologic doctrines have remained the cardinal references in the medical field up to the Renaissance, and the Hippocratic–Galenic techniques have been used in the orthopaedics field up to the XVIII century.

## The Origins of Western Nasal Surgery: Hippocrates and the Nasal Fractures Surgery

In the Hippocratic *De articulationibus,* we can find several descriptions of the system of bandages used to repair the fractures of the nasal septum and cartilages. Hippocrates, like Galen in its “*Commentaria*”, expresses his disagreement with the method used by some surgeons consisting in using only a bandage system to traction with a gauze the fractured part of the bone. This not only because the nose could deviate, but also because it could develop an extensive bony callus that could cause disfigurement and even respiratory disfunction. He agrees with the use of this type of bandage system only in cases where the tissues have been lacerated unilaterally up to the bone.

**Fig. 2:**
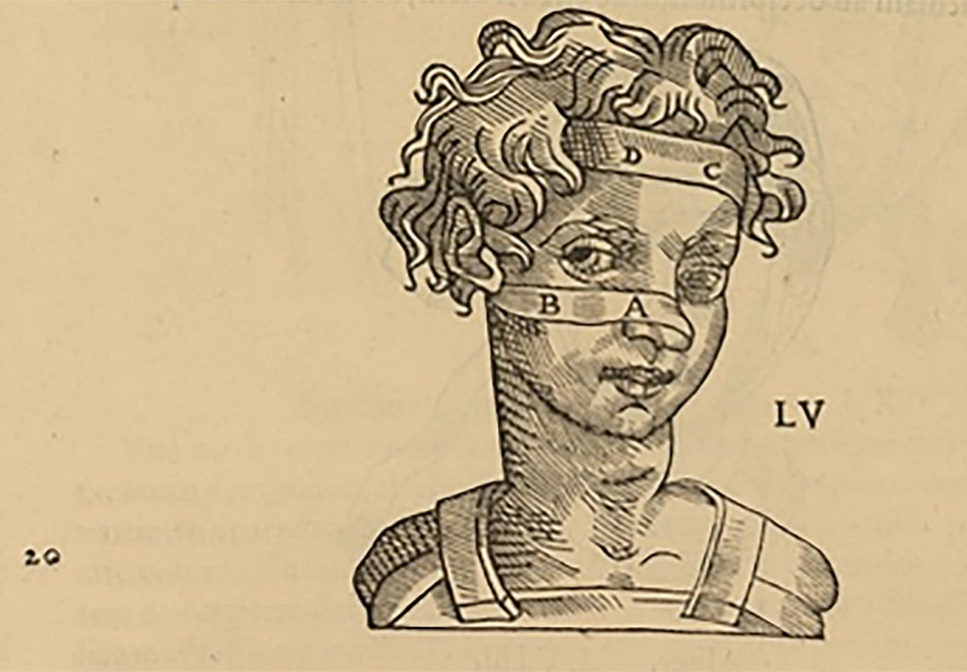
Example of nose bandage from an ancient illustration (see text). In Hippocrates, Galenus, Oribasius (ed. Guidi G.), *Chirurgia è graeco in latinum conversa, Vido Vidio florentino interprete*. Luceciae Parisiorum, Excudebat Petrus Galterius, 1544, p. 439. (Source: gallica.bnf.fr / Bibliothèque nationale de France)

In general, introducing a “primitive” idea of “splinting”, he suggests to wrap the nose completely with a single-waxed bandage that is tied in the back of the head without excessive pressure, as to create a forerunner of taping and plaster casts used today after osteo-cartilaginous humpectomy. Going more into details, he suggests to apply a small bandage soaked in *“agathoi puroi”*, literally “flower of flour”, which means fine flour, incense powder and Arabic gum (Fig. 2).

When the fracture leads to a depression of the nasal septum, Hippocrates suggests to realign the bone by inserting a spatula into the nostrils until the shape of the nose is restored and then introduce packings, or gauzes, or a piece of Carthage leather, into the nostrils, quite similarly, if not for the materials, to modern nasal packing. If the fracture involves the nasal root and dorsum, it is necessary, according to Hippocrates, to work only with the fingers, both from inside and from outside the nostrils, to realign the fracture fragments, without packings, which would cause excessive pain. The physician should repeat this operation for several days, with the aid of a woman with slender fingers or even a child working from the inside of the nostril, while the doctor works from the outside. Only later, if the nose shows signs of deviation, packings can be used [6]. Even today, some fractures of the nasal root, which are difficult to reduce, are often treated with secondary surgery and with the aid of modern imaging technology for the correction of residual deformities.

## Galen Between Past and Future: The Importance of Post-Traumatic Nose Dressings, “Fixation” and “Tension Free” Skin Closures

**Fig. 3:**
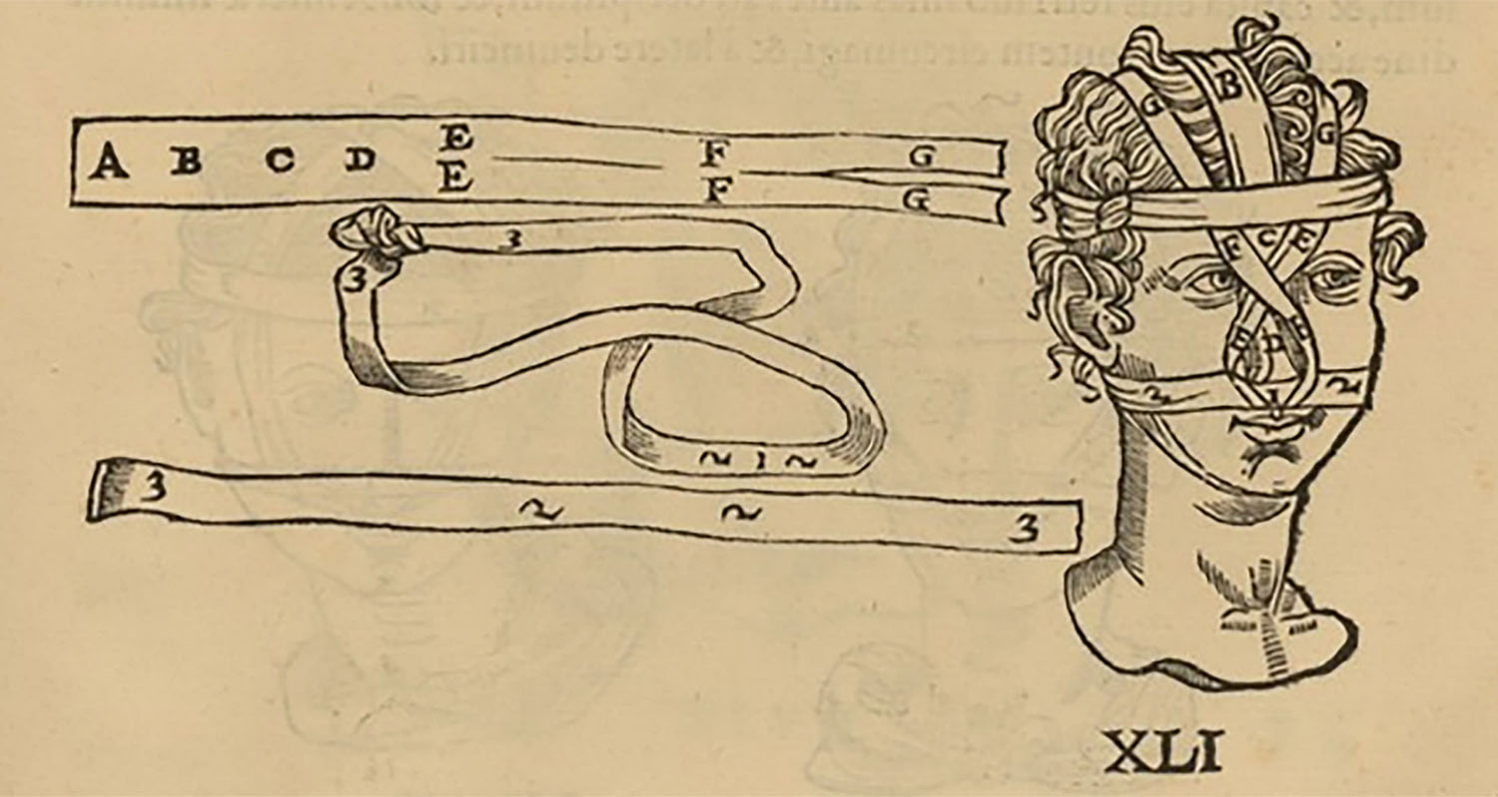
Bandages and their positioning on the nose from an ancient book illustration (see text) In Hippocrates, Galenus, Oribasius (ed. Guidi G.), *Chirurgia è graeco in latinum conversa, Vido Vidio florentino interprete*. Luceciae Parisiorum, Excudebat Petrus Galterius, 1544, p. 433. (Source: gallica.bnf.fr / Bibliothèque nationale de France)

The most interesting texts to understand every kind of bandage, and how to apply them, including those used for the wounds and the fractures of the nose, are the Hippocratic *De fascis* and its Galenic *Commentaria* (Fig. 3). Even if they do not describe the surgical procedures, they explain in detail all the different techniques to wrap the fractured nose, depending on the type of fracture. Some printed editions of their works in the Early Modern Age include images of the bandages used.

**Fig. 4:**
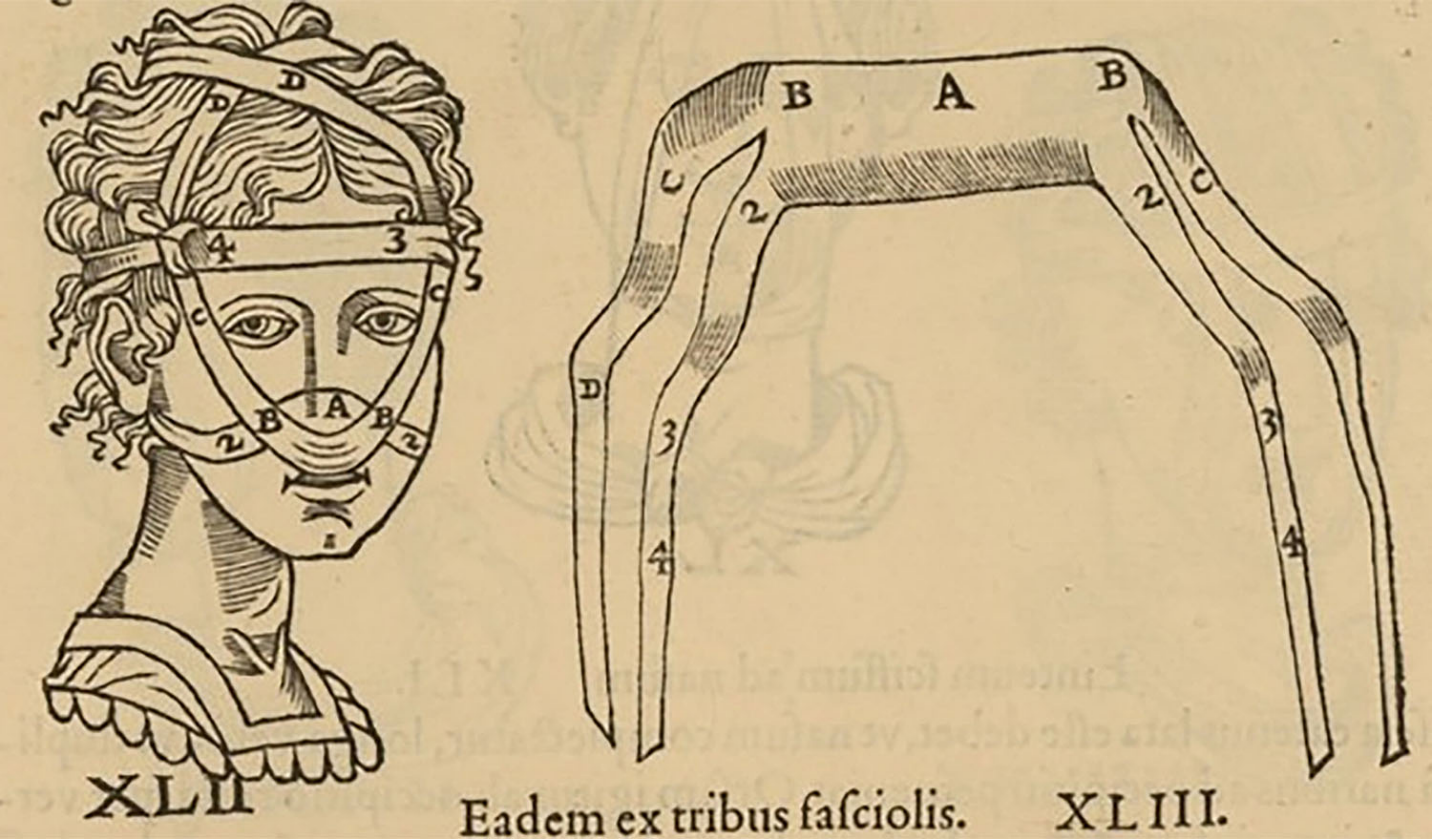
Bandages and their positioning on the nose from an ancient book illustration (see text). In Hippocrates, Galenus, Oribasius (ed. Guidi G.), *Chirurgia è graeco in latinum conversa, Vido Vidio florentino interprete*. Luceciae Parisiorum, Excudebat Petrus Galterius, 1544, p. 434. (Source: gallica.bnf.fr / Bibliothèque nationale de France)

**Fig. 5:**
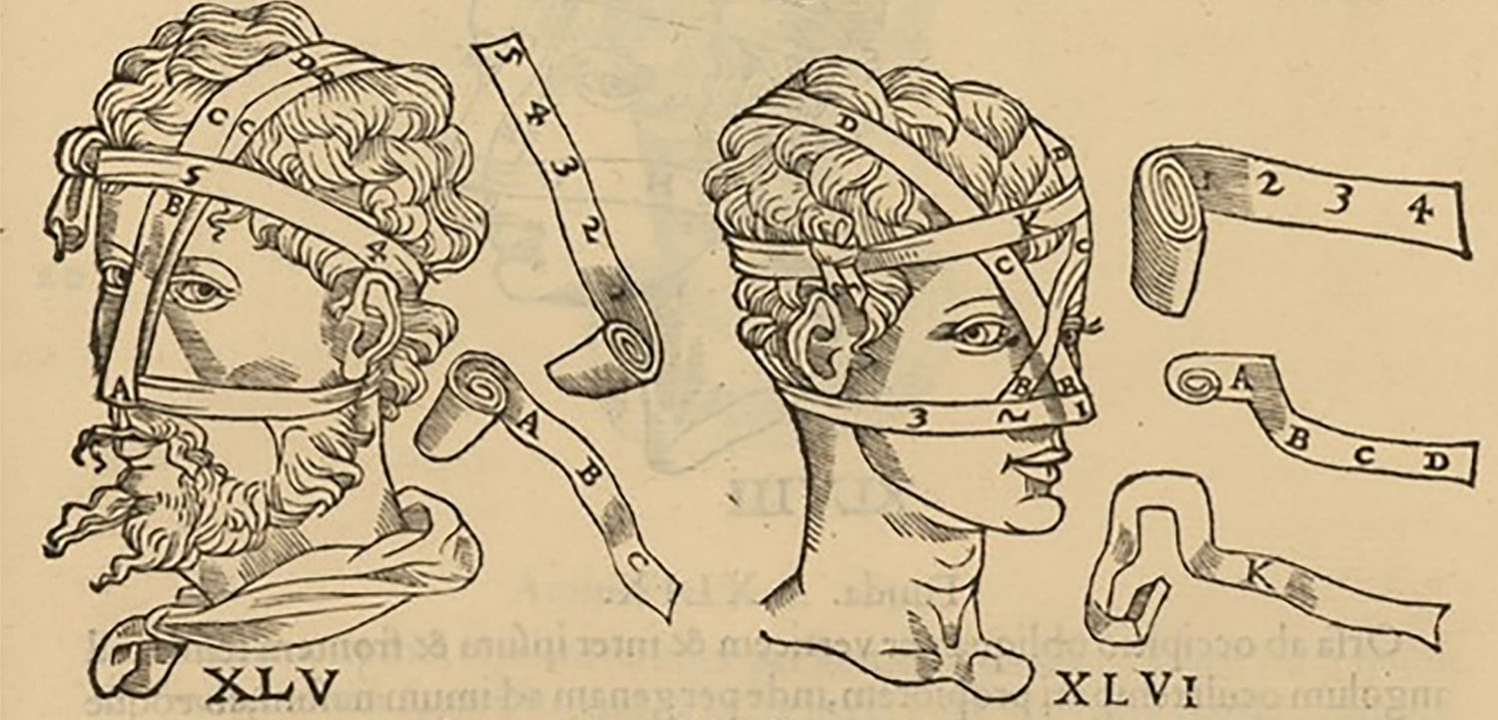
Bandages and their positioning on the nose from an ancient book illustration (see text). In Hippocrates, Galenus, Oribasius (ed. Guidi G.), *Chirurgia è graeco in latinum conversa, Vido Vidio florentino interprete*. Luceciae Parisiorum, Excudebat Petrus Galterius, 1544, p. 435. (Source: gallica.bnf.fr / Bibliothèque nationale de France)

According to Galen if the cartilage is depressed, or if despite manual interventions the nasal pyramid becomes deviated, a special bandage system should be applied, consisting in: gluing on the outside of the depressed area a strip of Carthage leather that pulls the part upwards or sideways, passing it behind the ears and finally tying it to the back of the head, or gluing it directly on the forehead [7] (Figs. 4, 5).

The Hippocratic–Galenic methods remain the model of reference also in Roman medicine, adopted and described also by A. Cornelius Celsus (Rome or Gallia, 1st century A.D.), author of the “De Medicina”, one of the most important texts to reconstruct the history of Roman medicine, rich in descriptions of surgical instruments, and surgical techniques. In his *De Medicina,* he describes some procedures for both facial deformities and wounds of the mouth, nose and ears. According to him, only small mutilations of these three organs could be treated, since interventions on severe mutilations could lead to further deformations the face.

The most interesting piece from Celsius is undoubtedly the description of the procedure for exeresis of cutaneous lesions of the nose: “*in case of nose amputation, you should create a square around the defect with lateral linear incisions then, starting from the corners of the square, a flap can be raised, advanced and sutured to cover the defect. If after this procedure the edges of the lesion cannot yet be closed, two other crescent shaped incisions can be made facing the previous ones, only through the superficial part of the skin, to allow tension-free closure of the wound. Sometimes the area in which the skin has been separated and pulled is left deformed. In this case, the deformed part is excised , leaving the rest intact.”*

It is extremely interesting to note the explicit reference to what is considered today one of the strongholds of plastic surgery, namely the necessity to apply minimal tension on skin flaps during inset.

On this topic the author continues saying that the skin should not be pulled from the lower part of the nose, nor from the margins of the nostrils, but from the central area of the nose, namely the nasal dorsum and radix. If the nasal cartilage protrudes trough the wound, it can be cut off and sutured once the margins have been approximated [8].

In this paragraph, another extremely modern concept is the resection of cartilaginous deformities in lesioned areas or in areas of distribution of tensile strength to improve scarring quality and to avoid distortion of contiguous anatomo-aesthetic areas. It is also possible to appreciate a basic concept of skin tension lines (editor’s note: the modern Langer’s lines).

In the chapter dedicated to fractures, Celsus explains how to intervene in the case of damage to the nasal cartilages and/or the nasal septum. When the cartilage is broken, it must be raised gently with the fingers in order to realign it and a special pad must be inserted into the nostrils. This pad consists of either a tiny strip of leather wrapped in interwoven threads, or a pen soaked in rubber or wax and wrapped in a thin layer of leather. If the cartilage is broken on one side only, a packing, larger than the defect, is inserted only on the injured side to avoid residual nose deviation, then the doctor should bandage the nose with a soft cloth coated with flour and incense soot, which is pulled up behind the ears, and tied together on the forehead. After 14 days, the pad can be removed using hot water.

In case of nasal bones fractures, the surgeon should reduce the fracture with his fingers as best he can and should fill the nostrils with packings as for the rupture of the cartilage (both nostrils in case of frontal fracture, or one nostril only in the case of lateral fracture). Then, the "waxing" is applied, which is a sort of plaster cast composed by bandages soaked in different natural substances with soothing and anti-inflammatory properties (wax, rubber and balms). The “waxing” should be tightened to avoid the formation of an hypertrophic bony callus. Even in case of comminuted fractures, the surgeon should slowly reconstruct the bony set-up of the nose, whilst for displaced fractures, where there are bony fragments, which cannot be reduced and may damage the skin, the surgeon should incise the skin and remove them with tweezers [9].

The Celsian technique is, in some parts, surprisingly advanced when considering that it was described in such remote times. Furthermore, the great attention paid to the bandaging technique shows that, at least empirically, he had already noticed the necessity to immobilize and compress bony fragments to allow effective healing.

## Eastern Roman Empire: The “Less Known” Byzantine Inheritance

The Celsian method was later taken up and further extended by Antillus (Greek physician active in Rome, II century A.D.), whose works were received only in the form of fragments reported by other authors, including Oribasius (Pergamon, Greece - now Turkey-, 325 – 403 A.D.). From indirect sources, we know that Antillus had precisely described techniques to repair facial wounds and to reconstruct the nose [10].

The Eastern Roman Empire represents a place and a moment of important cultural exchanges, of transmission, re-elaboration, and implementation of scientific knowledge, especially in the medical field. Among the authors of reference for Byzantine medicine there is Oribasius, author of a monumental work of compendium and extension of Greek–Roman medical literature. Greek–Roman doctrines in this period were systematically organized in thematic treaties of pathology, surgery, pharmacology, diagnostic, and many new surgical instruments and techniques were described.

## The Western Inheritance and Evolution of Oribasius: From Dressings to Specialized Surgical Instruments and Aesthetic Nose Surgery

Concerning rhinoplasty, Oribasius clearly learned a lot from the ancient *auctoritates*: in fact for treatment of nasal fractures he describes a bandage system which reminds the Celsian method. This consists in wrapping the entire nose in a circular fashion and tying the bandage on the nape of the neck. The bandage used must be soaked, in the part that surrounds the nose, with flour, gum and incense, if available, or, otherwise, honey and/or resins.

If there is a fracture that occludes or deviates the nostril, Oribasius says that it should be fixed operating from the inside of the nose with the fingers until the bony fragments are repositioned and, when necessary, long probes that can reach the injured area can be inserted up to the “sinuses of the septum”. In this description, we can notice that a new concept was starting to emerge, namely the importance of the aid of increasingly specialized surgical instruments, which allow to reach areas otherwise inaccessible, allowing to enhance the technical skills of the surgeon.

The author then offers a detailed descriptions for the treatment of various pathologies of the nose, including techniques to aesthetically improve the results of fractures that have not been well reduced.

In the treatment of "*egilope*" and "*anchilope*" that affect the lateral portions of the nasal bone (or, in current terms, fistulas of the lacrimal sac-dacryocystitis), or inflammations involving the upper part of the nasal septum (likely the authors refers to inflammatory pathologies of the nasal and paranasal sinuses), Oribasius proposes a special system of bandaging: a band of about a finger of width is fixed from the region of the *bregma* passing across the forehead to the sides of the nose and then descending along the margins of the lips, of the chin, under the neck until it can finally be tied at the level of the occiput. If the nose has suffered an excessive depression, laterally or caudally, a lint should be introduced to support the area and a piece of wool soaked in egg white should be placed on top of the nose. He approves the Hippocratic technique of fracture stabilization with a strip of Carthage leather, but prefers what he considers a better dressing system consisting in a bandage of about two fingers in width from the *bregma* to the forehead, turning down under the nostrils and then back towards the occiput [11].

Lastly, he also describes the system adopted by Heliodorus (Egypt, I-II century A.D.) in the case of displaced fractures [12]. This consists in the application of medicinal plasters and tablets (i.e. fabric plates which were also used as packings ) within the depressed part; then, a double perpendicular oblique bandage is used, which starts from the bregma, and it is pulled towards the nose to compress it and then passed along one side of the lips around the neck and to the occiput, from here it is pulled again over the nose and is brought back behind the head taking the same path contralaterally. [11].

## A Voice Out of the Chorus: Paulus of Aegina

Another important exponent of the Byzantine medicine is Paulus of Aegina (Greece, VII century A.D.), who, on rhinoplasty, refers directly to Hippocrates, but he also points out that many surgeons of his time did not fully agree, especially in the traumatological treatment. For example, he says that if the fracture is comminuted *“it is not enough to apply poultices, but the surgeon must incise, or widen, the wound, extract the bony splinters with hair scissors, suture and apply agglutinative drugs, such as the "lemnisci"* (i.e. a ribbon originally made of a thin vegetable membrane, then of wool); *lead cannulae* (forerunners of modern drainages) *are then inserted into the wound to purge and prevent excrescences from forming* “[13]. Back then in the pre-antibiotic era, even more than today, the thick and sebaceous skin of the nose is used to represent a challenge in achieving healing free from complications.

Byzantine doctors systematized the ancient medical knowledge in the form of specialized medical treaties that described new techniques to treat nasal injuries and new surgical instruments. Moreover, they made a remarkable work of translation of the ancient and contemporary Greek and Roman texts into the local languages (in particular Armenian and Syriac), thus allowing the fruition and diffusion of the Western medical knowledge in the Eastern countries, especially in the Arab world, where the text of 'Sushruta Samhita' has been translated in Arabic [14].

## Conclusions

This examination, though brief, represents an interesting testimony of how the history of rhinoplasty in ancient times precedes and is closely connected to the history of modern plastic surgery. Plastic surgery has always been originally considered a "war surgery", connected to trauma, and the "act of wound dressing" has always been an integral part of the surgical act in this discipline. The bandages, instruments, ointments described by the ancient authors of the Roman and Byzantine epochs seem to anticipate the modern treatises of wound healing and trauma care.

Moreover, through the History of Rhinosurgery, this paper, further completing the published research of authors [15], aims to highlight how Western medical knowledge is made up of the ensemble of cultures, which are apparently distant (from Pergamon, Alexandria, Byzantium, Amida, Baghdad, Toledo, Montpellier, Salerno) and different from each other, but deeply merge themselves in a truly universal and transcultural knowledge: the Medical knowledge. Never before this theme has been as topical as it is today: without sharing, without acceptance, without interpenetration, there is no scientific community, no scientific knowledge, no scientific progress.

Finally, once again we can abstract the teachings of the history of medicine which testifies how human culture, especially medical science, is universally shared by peoples and is the result of “trials and errors”, terms dear to modern epistemology, confirming how, to quote the words of Friedrich Nietzsche, *“Wer einst fliegen lernen will, der muss erst stehn und gehn und laufen und klettern und tanzen lernen: - man erfliegt das Fliegen nicht!”* [16] (He who would learn to fly one day, must first learn to stand and walk and run and climb and dance: one cannot fly into flying).
