# Correction to: The Medical Historical Cultural Foundations of Western Nasal Surgery from Ancient Greece to the Middle Ages

**DOI:** 10.1007/s00266-023-03272-8

**Published:** 2023-02-16

**Authors:** Silvia Marinozzi, Riccardo Carbonaro, Daniela Messineo, Edoardo Raposio, Luca Codolini, Giuseppe Sanese, Valerio Cervelli

**Affiliations:** 1 Department of Molecular Medicine, Unit of History of Medicine and Bioethics, “Sapienza” University of Rome, 34/a Viale dell’Università, 00161 Rome, Italy; 2 International Medical School, “Tor Vergata” University of Rome, Via Montpellier 1, 00133 Rome, Italy; 3 Department of Plastic and Reconstructive Surgery and School of Specialization on Plastic, Reconstructive and Aesthetic Surgery, School of Medicine and Surgery, “Tor Vergata” University of Rome, Via Montpellier 1, 00133 Rome, Italy; 4 Department of Radiological Sciences, Oncology and Anatomo-Pathological Science, “Sapienza” University of Rome, 31/33 Viale dell’Universitá, 00161 Rome, Italy; 5 Plastic Surgery Division, Department of Surgical Sciences and Integrated Diagnostics-DISC, University of Genoa, Largo R. Benzi 10, 16132 Genoa, Italy; 6 Unit of Plastic Surgery, “Sapienza” University of Rome, Viale del Policlinico 155, Rome, Italy; 7 PhD School on Medical-Surgical Applied Sciences-Plastic Regenerative research area, School of Medicine and Surgery, “Tor Vergata” University of Rome, Via Montpellier 1, 00133 Rome, Italy; 8 Private Practice, Rome, Italy; 9 University of Milan, Milan, Italy

**Correction to: Aesth Plast Surg** https://doi.org/10.1007/s00266-022-02989-2

This article was updated to correct the inversion of the authors’ given and family names.

